# The Gut-Skin Microbiota Axis and Its Role in Diabetic Wound Healing—A Review Based on Current Literature

**DOI:** 10.3390/ijms23042375

**Published:** 2022-02-21

**Authors:** Bharati Kadamb Patel, Kadamb Haribhai Patel, Ryan Yuki Huang, Chuen Neng Lee, Shabbir M. Moochhala

**Affiliations:** 1 Department of Surgery, National University of Singapore, Singapore 119228, Singapore; surbkp@nus.edu.sg (B.K.P.); surlcn@nus.edu.sg (C.N.L.); 2 School of Applied Sciences, Temasek Polytechnic, Singapore 529757, Singapore; kadamb@tp.edu.sg; 3 Canyon Crest Academy, San Diego, CA 92130, USA; rhuang53@gmail.com; 4 Department of Mechanical and Aerospace Engineering, University of California, San Diego, CA 92093, USA; 5 Department of Pharmacology, National University of Singapore, Singapore 117600, Singapore

**Keywords:** microbiota, dysbiosis, probiotics, diabetes, wound healing

## Abstract

Diabetic foot ulcers (DFU) are a growing concern worldwide as they pose complications in routine clinical practices such as diagnosis and management. Bacterial interactions on the skin surface are vital to the pathophysiology of DFU and may control delayed wound healing. The microbiota from our skin directly regulates cutaneous health and disease by interacting with the numerous cells involved in the wound healing mechanism. Commensal microbiota, in particular, interact with wound-repairing skin cells to enhance barrier regeneration. The observed microbes in DFU include *Staphylococcus*, *Streptococcus*, *Corynebacterium*, *Pseudomonas*, and several anaerobes. Skin commensal microbes, namely *S. epidermidis*, can regulate the gamma delta T cells and induce Perforin-2 expression. The increased expression of Perforin-2 by skin cells destroyed *S. aureus* within the cells, facilitating wound healing. Possible crosstalk between the human commensal microbiome and different cell types involved in cutaneous wound healing promotes the immune response and helps to maintain the barrier function in humans. Wound healing is a highly well-coordinated, complex mechanism; it can be devastating if interrupted. Skin microbiomes are being studied in relation to the gut-skin axis along with their effects on dermatologic conditions. The gut-skin axis illustrates the connection wherein the gut can impact skin health due to its immunological and metabolic properties. The precise mechanism underlying gut-skin microbial interactions is still unidentified, but the immune and endocrine systems are likely to be involved. Next-generation sequencing and the development of bioinformatics pipelines may considerably improve the understanding of the microbiome-skin axis involved in diabetic wound healing in a much more sophisticated way. We endeavor to shed light on the importance of these pathways in the pathomechanisms of the most prevalent inflammatory conditions including the diabetes wound healing, as well as how probiotics may intervene in the gut-skin axis.

## 1. Introduction

We dwell in a microbiome cosmos, comprising thousands of bacteria thriving both in and out of our bodies [1]. Bacteria, viruses, fungi, protozoans, and many other life-forms are now all categorized as microbes. Remarkably, these various microorganisms have survived on Earth for hundreds of thousands of years, a precursor to humans [1]. Bacteria, viruses, fungus, arthropods, and protozoa all reside in our digestive tract, which harbors approximately 100 trillion microorganisms. The microbiome, which has now been deemed a virtual organ, controls human systems [2]. Incredibly, these microorganisms reside within our bodies before we are born, live in a symbiotic association with us until we die, and safeguard us all from the spectrum of ailments. In addition to metabolizing nutrients, the intestinal microbiota serves another important role in health [3].

Adult humans have a diverse microbiota, with *Firmicutes* and *Bacteroidetes* representing 70% of the overall microbiota. *Proteobacteria*, *Verrucomicrobia*, *Actinobacteria*, *Fusobacteria*, and *Cyanobacteria* are some of the other microbial species encountered [4,5].

In fact, in every healthy adult individual there is indeed a heterogeneity in intestinal microbiota diversity between both the intestinal lumen and the mucosal surface [6]. *Bacteroides*, *Bifidobacterium*, *Streptococcus*, *Enterobacteriaceae*, *Enterococcus*, *Clostridium*, *Lactobacillus*, and *Ruminococcus* are among the most prevalent genera discovered in the intestinal lumen. *Clostridium*, *Lactobacillus*, *Enterococcus*, and *Akkermansia*, on the other hand, are by far the most common microbes on the mucosa-associated interface of a human gastrointestinal tract [7]. For its accessibility toward the intestinal mucosa and the accompanying mucosal immune system, the mucosa-associated microbiota plays a key role in preserving host cellular homeostasis or provoking inflammatory responses [8].

Microorganisms colonize the mucosal surfaces of the gastrointestinal tract, especially in the large intestine, and communicate with them in healthy adults. That is how microbes might effectively contribute, mostly in management of a variety of essential physiological functions. To begin with, they were found to play a role in the synthesis of vital nutrients, drug metabolism, and vitamin formation. Microbes additionally synthesize Short-Chain Fatty Acids (SCFAs), including such butyric acid, through fermentation of food, which does have nutritional benefits on the gastrointestinal tract epithelium [9]. Because of their antigenic traits, gut flora seems to be well known for their ability to stimulate the host immune system. It has been theorized that interconnections among our gut microbiota, gut mucosal cells, and the mucosal immune system promote a beneficial microbial community habitat that aids in minimizing the proliferation of pathogenic microorganisms [10] and, therefore, limits the colonization of the foreign pathogens in the intestinal tract [11,12,13]. The diversity of intestinal microbes and higher bacterial bio-diversity, often referred as eubiosis, characterizes healthier individuals’ gut microbiota. Bacteria are the most common microbes therefore in ecosystem, and that they are absolutely anaerobic and extremophiles. *Firmicutes* and *Bacteroidetes* are by far the most predominant bacterial phyla, representing around 85–90% of all microbes; *Actinobacteria* and *Proteobacteria* are much less prevalent, accounting for up to 10% of total microorganisms [14]. When commensal bacteria outnumber pathogenic bacteria, this is the condition by which this ecosystem balancing is disrupted due to either antibiotic usage, motility difficulties, nutrition, or host genetic predisposition; the resultant situation is called gut dysbiosis [15]. This is highlighted by a diminution in the diversity of diverse bacterial species, as well as an increase in the abundance of pathogenic bacteria, causing the loss of microbiome physiological functions [15,16]. This observed phenomenon is called leaky gut, and sometimes it enables bacterial migration and performs a central role in the growth of gastrointestinal and systemic disorders in humans due to a change in microbial species [13,17].

Our skin, like that of the microbiota of the human gastrointestinal tract, is a habitat of millions of bacteria, fungi, and viruses. Furthermore, evaluation of the human microbiome employing various technologies, namely culture-independent, high throughput 16S rRNA gene sequencing, have established that diversity of the gut microbiome at different locations on the body and at varying depths of the skin [18]. On human skin, there are around 1200 different types of bacterial species. *Propionibacterium*, *Staphylococcus*, and *Corynebacterium* are the most prominent taxa encountered on the skin [18]. There is also some evidence that even more bacterial species such as *Cutibacterium*, *Staphylococcus*, *Micrococcus*, *Bacillus*, *Roseomonas* and *Paenibacillus* are ubiquitous and functional on the skin microbiome [19].

The main known important functions of skin microbiome are in immune interactions, wound healing, colonization resistance, and various skin disorders. Although much remains still to be discovered and to be identified in terms of the host pathways that are influenced by skin microorganisms and the higher-level skin properties impacted through these microbe-host interactions [20]. There are reports available postulating the vital role of the microbiome in various skin disorders. Recently, the available research resources through various scientific discoveries have described that not only different skin disorders that play a significant role in altering our gut microbiome in humans but existence of various skin diseases are associated with an altered gut microbiome [21].

Type II diabetes, a metabolic ailment defined by elevated blood glucose levels, is among the world’s fastest-growing chronic illnesses. Clinical scientists and physicians projected that the world prevalence of this condition will indeed surge from 382 million in 2013 to 592 million by 2035 [22]. Moreover, it is claimed that nearly 10% of adult natives globally have diabetes or are at a high risk of developing it. Diabetic foot ulceration is by far the most typical severe complication in people with diabetes, and this is influenced by a multitude of conditions include peripheral vascular disease, sensory motor, and autonomic neuropathy, among many others [23].

Live microorganisms have been used in bioactive agents such as probiotics, when administered in the correct dosage to the appropriate person, can also have numerous health benefits [24]. As a result, any intervention that alters the gut microbiome has the potential to improve the metabolic control of patients with type 2 diabetes, at least to an extent [25]. As more than just a reason, these bioactive agents may well be vital in the treatment and prevention of diabetes. Probiotic intake had already previously shown to enhance intestinal health, ameliorate lactose intolerance signs, impede pathogenic bacterial growth, start producing Short-Chain Fatty Acids (SCFA), revise the gastrointestinal (GI) tract, sustain PH, activate the immune system, as well as minimize the risk of various types of diseases. Modifications in the gut microbiota produced by probiotic supplementation may also have anti-diabetic effects, as according preliminary report [26]. Probiotics potentially may play a significant role in metabolism, immunomodulation, wound healing, and a variety of inflammatory and infectious disorders and diseases, as per a wealth of evidence. Regardless of their physical differences, the skin and gut have a lot of common physiological features and undertake several functions via cross-talk between both the two compartments [27]. Furthermore, probiotics can also be used as contemporary therapeutic strategies or as supplements to routine therapeutic approaches in the treatment of a wide range of human diseases. To this day, more research data to endorse the clinical applications of oral or topical probiotics in managing skin and gut-associated diseases are required to support the clinical trials [27]. The microbial bio-burden is known to contribute to wound infection healing impairment. Over 50% of DFUs are infected once they present [28]; however, the infections are hard to diagnose due to the diminished or absent clinical signs in DFUs induced by peripheral neuropathy and/or vascular disease [29]. As encountered in diabetic patients, a most therapeutic challenges affiliated with DFU are deferred wound healing and diabetic foot infections [30]. It has been generally speculated that due to the rising issues with anti-microbial drug resistance among people with DFU worldwide, clinical scientists have also strongly advised that the amalgamation of probiotic use with current therapeutics would become exhilarating and meaningful in diabetic patients. Because probiotics can boost the immune system and have anti-inflammatory qualities, they might well assist in the healing of wounds in DFU patients [31]. Probiotics could also certainly assist to metabolic control. As a result, a creative approach, such as probiotics consumption, could indeed eliminate harmful microorganisms and might even improve the healing process [31].

Derived from empirical breakthroughs concerning diabetic foot ulcers (DFUs), it is indeed straightforward that all these wounds have a polymicrobial nature which ends up going far beyond identification capabilities of bacteria previously used by culture methods for chronic wounds [32]. As a matter of fact, there is a need to better understand existing therapeutic strategies to cure or prevent wounds, not just to safeguard patients and family members, but rather to protect the economic systems of healthcare systems around the world. The main objective of the review is and see if there are any variations in the skin microbiome of diabetics, as well as any representatives of a skin microbiome linked to diabetes-related chronic wounds. Probiotics are considered to be safe, but much more investigation is necessary to demonstrate safety precautions in immune-compromised individuals. Recent research on the gut-skin axis emphasized the significance of probiotics in gut microbiota, skin homeostasis, as well as wound healing [27]. Clinicians will face additional problems understanding, describing, and characterizing the wound microbiota from beginning to end. By holistically assessing the literature, this could contribute to the development of more effective and strategic therapies of microbial administration in existing medical therapeutics for diabetic wound healing in clinics and hospitals, such as probiotic topical creams and bacteriophage therapies for diabetic wound healing in upcoming years. This review attempts to provide a qualitative summary of how this field has nudged the boundaries of our understanding of the gut-skin axis and the influence of microbiota on health and disease, as well as what remains to be acknowledged in order to fully realize the prospects of microbiota-based therapies useful for treating diabetes foot ulcers.

### Diabetes Foot Ulcers (DFU)

Many studies mainly address the one primary condition in diabetic people that requires a significant amount of attention, called Diabetic Foot Ulcers (DFUs), which are common and one of the most severe complications of this disease. Diabetics are more likely to experience this problem due to its high frequency and impairment of the wound healing process; the lifetime risk of developing a foot ulcer for diabetics is estimated at 25% on average [33]. Nevertheless, peripheral neuropathy, peripheral arterial disease (PAD), and trauma are the most common factors associated with the development of foot ulceration. Furthermore, the appearance of impaired wound healing is the primary complication that results in the development of chronic wounds, which often lead to amputations. Diabetes is one of the most common causes of non-traumatic lower limb amputation, and 15–20% of all foot ulcers will ultimately require amputation [34]. Defects in microcirculation, which are often accompanied by peripheral neuropathy and peripheral arterial disease, contribute to the development of diabetic foot problems. In addition, several anatomical foot deformities, which impair loading across the plantar surface, have also been clinically associated with impaired wound healing of diabetic patients.

Moreover, diabetes alters the immune response and lowers resistance to infection in patients. Thus, diabetic wound infection further impairs healing through several mechanisms [33,35]. It is apparent that the wounds in the diabetic foot are difficult to heal, not easy to treat, and are highly susceptible to infection. With the advanced development of high throughput next-gene sequencing technologies accompanying the microbiome research, the possible correlations between the microbiome and the onset of Type 2 Diabetes Mellitus (T2DM) have become now more influential in the past few years. More specifically, the entire focus on microbiome profiling of patients with T2DM has begun to be evaluated to seek a new treatment option. Many available reports have revealed that the existence of microbial composition in T2DM patients is quite different from non-T2DM individuals [36].

Regarding the pathogenic role of bacteria observed in patients with DFU, there are currently two main hypotheses [37]. The specific bacterial hypothesis proposed that only a few species of bacteria within the heterogeneous polymicrobial biofilm are involved in the overall infectious process. Conversely, the nonspecific bacteria hypothesis (or community hypothesis) that concludes the composition of bacteria observed in a biofilm as a whole to constitute a functional unit and does not examine the role of individual pathogenic bacteria alone. This concept has led to using the term ‘‘functionally equivalent pathogroups’’ (FEP) [38]. Finally, it proposes that certain bacterial species, which are usually non-pathogenic, or at best, are not capable of maintaining a chronic infection when present alone, may co-aggregate symbiotically in a pathogenic biofilm and act synergistically to exacerbate chronic infections [39].

## 2. On the Horizon: Gut-Skin Microbiome Axis

Multiple research findings revealed that the gut microbiome performs a prominent role in several skin disorders. Not only is the skin microbiome altered, but also surprisingly many skin diseases are accompanied by an altered gut microbiome [21]. Several reports described the gut-skin axis, which shows the relationship between the gut microbiota and the skin. It is one of the most promising research areas currently targeting the microbiome of the skin, which plays a critical role in controlling the cutaneous processes critical to human health and disease [40]. Additionally, the overall constitution of various microbial communities observed on the skin primarily depends on the physiology of the different sites of our skin, with alteration in the relative abundance of bacterial taxa associated with moist, dry, and sebaceous microenvironments. It is profound that some of the lipophilic *Propionibacterium* species mainly influenced the sebaceous sites. Microbes that prefer moist, humid environments, such as *Staphylococcus* and *Corynebacterium* species, were more prevalent in areas that had a high moisture content, such as elbow bends and feet [41].

A skin microbiome is a group of complex communities that contain different species of microbes. It is a highly dynamic microbial community that helps maintain an interdependent relationship with the host [42]. In addition, the human gut is microbially inherited, and the skin barrier shares striking common features with gut microbes. The countless purposes and functions of gut and skin microbes are incredibly similar, as discovered through research on the microbiome over the past few years. Both are highly innervated and vascularized. They are essential for various immune and neuroendocrine functions and are therefore important for the assembly of the gut–skin axis [42]. 

The gastrointestinal tract’s inner surface and the outer surface of skin cells are layered by epithelial cells (ECs) that are directly connected to the exogenous environment. The epithelial cells help to maintain an important connection between both the internal body and the external environment; thus, they act as first line of defense, preventing various microbes from entering the gastrointestinal tract [43]. There is evidence to support the existence of a bi-directional association between the skin and the gut that also combines the possible connection between gastrointestinal health and skin homeostasis and allostasis in humans [44]. Moreover, the immune system is continuously primed to differentiate between harmful and beneficial compounds in our bodies; according to some reports, there is a profound bidirectional relationship [45].

Microbes inherited in our gastrointestinal tract play an important role in our daily lives; the gut and skin have so many similar characteristics. These highly vascularized, well-perfused, and intensively innervated structures are colonized with distinguishable microbial populations and represent as vital contact organ systems by which mammalian organisms communicate with their surroundings. Further to that, these relatively small populations of microbes are complex immune and neuro-endocrine organs that perform an important role in the immune and endocrine systems of the entire body. As a consequence, both from an evolutionary and medical standpoint, their proper functioning is critical for maintaining homeostasis and survival. The intestinal microbiota is the ‘virtual organ,’ with substantial immunological and metabolic implications. It has an effect on the several organ systems, including skin. Other studies have also suggested its involvement in skin health primarily due to modifying the immune system in humans [46,47,48]. 

The concept of the “skin-gut axis” has surfaced in past few years and has been an important scientific platform; nevertheless, pathobiological understandings are all still suffering from a lack. Although the pathways clarifying how well the gut and skin interact really are not precise, it is likely to be involved a complex interrelationship between the nervous, immune, and endocrine systems, as well as environmental factors [42,49]. A gut-skin axis communicates mostly through metabolites, the neuroendocrine system, diet, and the central nervous system, according to research hypotheses. Nonetheless, the gut-skin correlation is primarily determined by modifications in gut microbiota and their products, as well as indirectly by changes in the gut epithelium’s diet, which influences the intestinal flora and the skin [50]. The close interconnection between the gut and skin is beyond doubt. Perhaps, both the intestinal microbes themselves and their metabolic by-products govern the skin physiology. 

The theories underpinning how well the gut-skin axis functions are still being explored, though there are some concepts that have been recommended: (1) bacterial products as well as diet might very well modify the physiology of the intestinal epithelium, causing numerous secretory products which may circulate systemically to reach the skin; (2) neurotransmitters, hormones, and other biologically active chemicals derived from intestinal flora, including SCFAs, could function on receptor sites within the skin and it may effectively change the skin or alter the skin’s commensal microbes; and (3) ingested substances and chemicals, at first when absorbed, might indeed change the skin or alter the function [50].

## 3. Gut-Skin Microbiome Interactions in Complex Dermatological Diseases

The formation of an individual’s skin microbiota during the intrapartum period is now widely recognized. As a result, the mother’s mode of birth influences the skin’s overall microbial structure [51]. The microbiome is determined by that of the host genes, according to a recent metagenomic cross-talk study. The microbes construct a “holobiont” with the infant, which would be a combination of a host and microbial populations that imparts capacities to the hosts. This could represent an important role in the early life health [52]. In other words, some intestinal microbiota shapes and behaviors may be genetically linked in babies, and the microbiota, in turn, regulates host gene expression. The combination and association of the growing intestinal microbiota with host genetics could therefore play a significant role in newborn early stages of development in customized intervention of vulnerable infants. The microbiome shapes host genes, and indeed the microbiota, in turn, influences host gene expression, as per recent research [53].

Different microbial populations colonized distinct epidermal niches, indicating that colonization is site-specific. The normal skin microbiota thrives on this feature [54,55]. The skin is a multifaceted barrier organ comprised of a symbiotic association among bacteria and host, particularly demonstrated by breakthroughs in microbiome research. The adaptive and innate immune devices transmit multiple signals which maintain the human skin in continuous interaction with them. The microbiota, which really is vital for the health of the skin, is monitored delicately through this mutualistic relationship. Continuous exposure to environmental exogenous and endogenous factors on the skin, on the other hand, may have a detrimental effect in this very well structure, culminating in pathological processes. In the absence of effective compensatory mechanisms, inflamed skin might arise [56]. 

The development of the human microbiome has received much interest in recent decades. Both gut and the skin are considered to somehow be extensively occupied with microbiota that comprises of a range of microorganisms. Human skin is expected to have 10^12^ microbes, whereas the stomach contains 10^14^ bacteria [54]. *Staphylococcus epidermidis*, *Streptococcus luteciae*, *Bacillus* sp., *Roseomonas mucosa*, *Paenibacillus* sp., *Micrococcus luteus*, *Corynebacterium* sp., and *Acinetobacter lwoffi* have been recognized as microbiome delegates from genera and species that prevalently dwell inside the aerobic environments of a skin surface4]. Consequently, the skin serves as that of the body’s largest and most visible barrier toward the outside environment. It is heavily populated with immune cells and extensively colonized by microbial cells, which stimulate the immune cells. It does have an impact on the host’s well-being [57]. As in areas of dermatology, skin disorders, as well as its association with and influence on the innate immunity, the cutaneous microbiome has received a great deal of attention in recent years. There seem to be studies have linked several skin diseases to a microbiome imbalance in the skin. However, understanding if whether altered skin microbiota is indeed a significant cause or a consequence of the skin ailment is challenging [57].

The immune systems, including adaptive and innate immune, modify the microbial composition; nevertheless, the indigenous microbiome can however regulate the immune system. The underlying mechanisms of how the gut microbiome impacts the immune system of a skin and vise—versa are currently being evaluated. Numerous skin disorders constitute comorbidities with the gut. Several earlier studies have described a clear communication and mechanism between commensal skin bacteria and host tissue in a variety of skin diseases. Microbial species including *Staphylococcus epidermidis* and *Staphylococcus aureus* have been found to induce important signaling pathways, resulting in a distinct modulatory innate immune system8]. Similarly, a cell wall component common to the *Corynebacterium* genus modulates an additional distinct immune system pathway, interleukin-23 (IL-23)-dependent inflammation [58,59]. Abnormal microbiome compositions, which are often characterized by a reduction in microbial diversity, have also been related to diabetes and various skin disorders in certain disease states [20]. Microbial communities that occur in wound tissue are difficult to pinpoint and are not really connected with cardinal symptoms of infection, further complicating wound healing prognostics [60]. 

Human skin (about 30 m^2^ in adults) and intestines (400 m^2^ of intestinal epithelium) have considerable interactions with the surroundings, leading to a high potential of pathogen attack [61,62,63]. These locations should safeguard themselves against infections through a variety of protective factors in conjunction to harboring tens of millions of commensal bacteria. The skin and gut both have typically refined, chemical, bacteriological, as well as an immunological barrier in order to avoid harmful pathogens aggregating. The epidermis and the intestinal tract, both active immune organs, have developed symbiotic relationships with commensal bacteria, culminating in extensive regulatory frameworks to preserve equilibrated homeostasis that actively supports commensal microbes while defending off invaders. Furthermore, epithelial cells of the skin and intestine generate antimicrobial proteins (AMPs) that behave as endogenous antibiotics in the battle against pathogens; these immune cells also keep monitoring these surfaces and it may promote wound healing processes [64]. 

Owing to its immunological and metabolic capabilities, the gut-skin axis helps to explain the overall relationship between both the gut and the skin that has a profound influence on the skin’s overall wellbeing [42]. Nonetheless, a precise cause-and-effect relationship between both the gut microbiome and diverse dermatologic pathologies should be documented. According to multiple studies, there is a connection between both of them as well as a variety of cutaneous diseases associated with GI disturbances and vice versa [65]. Furthermore, several of the cutaneous manifestations of inflammatory bowel disease may very well be associated with the degree of intestinal inflammation [66]. 

The possible framework for this outcome is that a gut imbalance provokes T-cell stimulation while also interrupting immunosuppressive cytokines as well as regulatory T cells (T reg cells), which seem to be responsible for ensuring microbiota tolerance [67]. As a result, there really is a pattern of chronic inflammation in the gut and on the skin, which cannot be self-regulated by the standard immune reaction. The gut is identified as one of the most important immune organs, with one of the most complex immune compartments, gut-associated lymphoid tissue (GALT). Peyer’s patches that are made up of structured lymphocytes and thus are known as the primary inductive sites of mucosal immunity are also an important component of the GALT. Dendritic cells in Peyer’s patches synthesize and induce IL-10, causing T helper cells to divide and proliferate. Cytokines and primed immune cells may be transported from Peyer’s patches to the skin via circulation, where they modulate immunity and enhance the defense mechanism, potentially providing a link in the gut-skin communications [46,67].

## 4. Mechanistic Insights into the Diabetic Wound Healing

**Figure 1:**
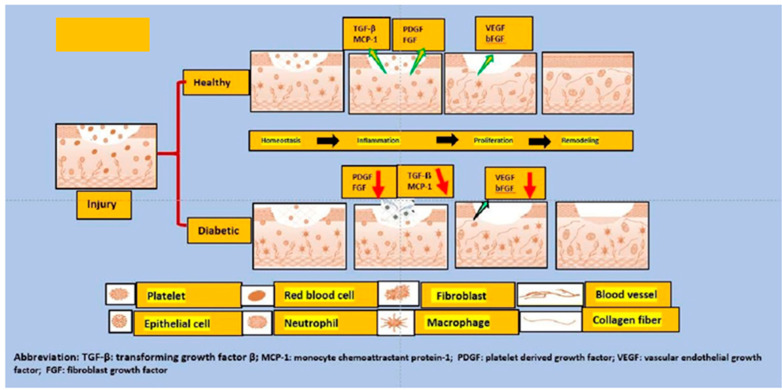
This figure depicts the differences between the normal and diabetic wound healing phases. When an injury occurs, the formation of various growth factors and cytokines such as transforming growth factor (TGF-) and monocyte chemoattractant protein 1 (MCP-1) from aggregated platelets begins. This is required for the formation of new tissue in the first stage. As a result, growth factors, keratinocytes, and activated fibroblasts start to form a new extracellular matrix and new blood vessels. Reduced levels of all growth factors and cytokines (such as TGF-), MCP-1, and growth and angiogenic factors (VEGF and PDGF) are major contributors to diabetic foot ulcer refractoriness. In short, wound closure is severely impaired in diabetic foot ulcer patients; additionally, reduced angiogenesis is observed due to the hyperglycemic phase, decreased migration of keratinocytes and fibroblasts, resulting in a deficient re-epithelialization; similarly, poor production of the extracellular matrix by fibroblasts contributes to the problem of deficient wound closure.

The hunt to understand the mechanism involved in wound healing has been ongoing in recent years, and it is now well hypothesized that the wound healing, a very complex development process, is physiologic and observed in a state when the integrity of the skin is almost damaged. Consequently, the barrier function of the skin is also damaged. This phenomenon may appear quite often because the skin is frequently exposed to specific external insults. There is a need to avoid systemic infections that drive a rapid defense mechanism [68]. The normal status of the skin can fully recover through physiological healing. However, it is claimed that only about a maximum of 70% of previous tensile strength is usually achieved [69]. The patient with diabetes does not follow a typical pattern for wound healing like an average individual, which is the major problem. Usually, in non-diabetic status, the dynamic process of wound healing comprises four different phases: hemostasis, inflammation, proliferation, and remodeling [70]. In the initial stage of wound healing, there is the involvement of cell repair that consists of the platelet activation, aggregation, and adhesion to the damaged endothelium-this overall helps maintain the hemostasis. Upon initializing this mechanism, the fibrinogen becomes the fibrin that forms the thrombus and a temporary extracellular matrix (ECM). A few other cells, including activated platelets, neutrophils, and monocytes, release some proteins and various growth factors, such as platelet-derived growth factor (PDGF) and transforming growth factor β (TGF-β), also participate [70]. In diabetes, hypercoagulability and a decrease in fibrinolysis are some of the observed changes in the hemostasis phase compared to normal subjects [71]. When an injury to the tissue occurs, initiation of the inflammatory process begins; neutrophils, macrophages, and mast produce various inflammatory cytokines, such as interleukin 1 (IL-1), interleukin 6 (IL-6), tumor necrosis factor-alpha (TNF-α), and interferon-gamma (IFN-γ). Several growth factors, including platelet-derived growth factor (PDGF), epidermal growth factor (EGF), and insulin-like growth factor 1 (IGF-1), the primary fundamental in the wound repair process, are also produced during this sequential mechanism of wound healing [72,73]. However, in patients with diabetes, a disequilibrium of these cytokines leads to a modification of wound repair mechanism [74] and pattern of altered cytokine release shows a decrease in their functionality, contributing to the susceptibility to wound infection in diabetes patient [75]. During the phase of proliferation and migration, the level of inflammation is reduced. Various processes begin at the lesion site causing the wound contraction; angiogenesis restores the supply of oxygen; extracellular protein (ECM) proteins form, including the collagens, fibronectin, and vitronectin. All these components are essential for cell movement further in addition to keratinocytes migration-this sequential phenomenon is very important for the tissue to restore its integrity and functionality [76]. In hyperglycemic patients, migration of fibroblasts and keratinocytes and their proliferative capacity is diminished. Thus, overall, abnormal cell migration leading to a deficient re-epithelialization of the diabetic wound, which affects the healing process [72,77]. Moreover, it was reported that in diabetes patients, a decrease in angiogenesis causes a decrease in blood flow [78]. The last phase of wound healing is called the remodeling phase, where synthesis of more significant than degrading collagen occur and replacing the provisional extracellular matrix (ECM)-which was initially formed by fibrin and fibronectin. This granulation tissue becomes more mature scar tissue that increased the wound resistance and ended in the formation of a scar [79]. What happens in diabetes patients is the altered function of fibroblasts, which is responsible for contributing to the defective closure of the wound. Although the entire mechanism has not been deeply investigated, it is believed that they do not respond to the action of TGF-β, as well as the aberrant production of the ECM [80]. The overall mechanism of wound healing is depicted in Figure 1 [81].

## 5. Microbiota Involved in Wound Healing

Normal wound healing takes place as a basic biological system inside the human body through four precise and highly programmed stages, hemostasis, inflammation, proliferation, and remodeling. For any wound to recover fully, only those four phases should happen, and in the right order and time scale. Normal skin wounds heal in approximately one to two months. It is indeed a natural, biological, and sophisticated process that occurs after a tissue injury and tends to involve blood cells, connective tissue, parenchymal cells, ECM, as well as soluble mediators such as cytokines and growth factors communicating each other during the wound healing mechanism [82]. Microbial colonization occurs in any and all types of wounds e.g., acute well as chronic, and there is a break in epithelial barrier which characterizes a wound impairs the factors that influence and constrain the microbial community at that site. A wound is be associated with the physical interruption in the integrity of the epithelium as well as the subsequent host immune response to fix this break. Any breach in the epithelial barrier impedes the events that shaped and confine the intestinal microbiota at around that site. Destabilization of the epithelium diminishes mucus or lipid production, distorts anti-microbial peptide representation and stimulates inflammatory cascades. Because mucosal surfaces are exposed to the environment, wounds allow non-indigenous microbes to colonize the site while also changing the forces that stabilize indigenous microbial colonization [83]. Over the last few years, considerable evidence on the human microbiome already reinforced the hypothesis that the ecological microbial community in/on humans is crucial in the host for maintaining the homeostasis, and also any internally or externally factors that cause dysbiosis of the skin commensal microbiome in diabetes patients also may prompt the shambles of immunologic stabilization inside the skin and therefore would empower the onset of varying skin diseases [41]. Furthermore, under various physiological conditions in a healthy individual, the observed mechanism of cutaneous epidermal repair is highly efficient; however, when this process stalls due to various external and internal factors in the host, the function of tissue deteriorates to reclaim structural and functional integrity, resulting in the formation of chronic wounds [84]. It is preferable to stabilize signaling factors, which include growth for effective wound healing; however, down-regulation of such factors contributes to the pathophysiology of DFUs. Additional factors known to promote wound healing delay in diabetes involve macro- and microvascular, neuropathic, immune function, and microbiome disturbances [85].

Much previous research investigated the implicated mechanism associated with wound healing, and recognized that the whole framework is, generally speaking, not straightforward. It involves an interaction between multiple cell types, primarily epidermal keratinocytes, neutrophils, and macrophages, and the inherited resident commensal microbiota of the gastrointestinal tract in humans. Wound colonization was observed in the later mechanism, accelerating wound healing by influencing the host’s innate immune system. Keratinocytes have expanded and migrated as part of the mechanism, and fibroblasts also exited and amassed the extracellular matrix (ECM) during this stage of wound healing. During the proliferation phase, angiogenesis takes place. During the remodeling phase of this entire mechanism, the extracellular matrix (ECM) reconstructs the appearance of scar formation, followed by the recovery of the epidermal skin barrier. The epithelial barrier will not fully heal when one of the phases is interrupted in just about any way, leading to the formation of a chronic wound. Furthermore, wounds provide a desirable moment of opportunity for microbiota to acquire access to the underlying tissues in order to colonize and grow further [86,87]. 

Scientists also expanded on the ongoing relationship between cutaneous and gastrointestinal microbes, claiming that any changes in local cutaneous and gastrointestinal microflora may positively or negatively impact wound healing via various pathways. One of these is that it primarily affects the host through the production of antimicrobial molecules and the regulation of the host’s inflammatory and immune response [88,89,90,91]. Furthermore, clinical assessment clearly shows that impeded wound healing is a strong predictor of mortality and morbidity in a considerable number of people with diabetes worldwide [92]. 

Microbes can also have an adverse effect on the wound healing process. Specific bacteria, such as *Staphylococcus aureus*, have been linked to wound infections and complications. More specifically, known microbes such as *Staphylococcus*, *Anaerococcus*, *Corynebacterium*, *Porphyromonas*, and *Streptococcus* are abundant in the chronic wound microbiota. [93,94]. In addition to cutaneous microflora, intestinal microflora influences wound healing by directly or indirectly attempting to influence a variety of healing factors including tissue oxygenation levels, blood pressure, inflammation, and the immune system [93]. Despite the high oxygen levels in chronic wounds, anaerobes such as *Fingelodia*, *Prevotella*, *Peptonipihlus*, *Peptostreptococcus*, and *Anaerococcus* have emerged as major threats [95]. 

## 6. Altered Microbiota in Diabetic Wound

Amputations of lower limbs due to diabetic foot ulcers accounted for 40–70% of all non-traumatic amputations, according to research findings. Foot ulcers occur prior in approximately 85 percent of all amputations in diabetics. When the ulcer progresses to its most complicated form, treatment becomes more difficult; in many cases, diabetic patients must be admitted to hospital [96]. Surprisingly, one of the findings looked at a significant difference in the abundance of microbial communities in the healthy skin of the foot and forearm of 30 diabetic patients versus 30 healthy people. The results revealed a statistically significant change in the microbial community as well as skin diversity in diabetic forearms but not in non-diabetic’s forearms. The phylum *Firmicutes* is more prevalent in non-diabetic foot skin, whereas *Actinobacteria*, specifically the species *Corynebacterium*, is more abundant in diabetic foot skin and has been linked to higher *Staphylococcus aureus* carriage rates [97]. 

Bacterial interactions on the skin’s surface play a critical role in the pathophysiology of diabetic foot ulcers (DFU). It is essential in the wound healing mechanism and may contribute to healing delays when there are numerous unfavorable conditions [98]. The host-microbe interface is frequently cited as a critical point in the development of wound infections. The clinical judgment, however, concluded that the observed number of pathogenic microbial species at this interface is lower when compared to the presence of many commensal bacteria. Furthermore, many of the species found in chronic wounds are commensals in healthy skin, and there are clear differences in the composition and diversity of the microbiota in diabetic foot ulcers (DFU) and healthy skin microbiota [99].

It is critical to place the recent acknowledgment of the microbiome’s impacts on health in an evolutionary context. With the advancement of microbiome research, various groups of scientists identified a possible link between altered microbiota and various diseases. However, it remains a mystery whether such changes are the cause or the result of various diseases, or whether various diseases cause an altered microbiota composition. The microbial composition of human skin is not always static; the presence and abundance of different types of microbes in skin wounds are primarily determined by the type of wound observed. However, it is known that the three major phyla identified in pressure ulcers, namely *Firmicutes*, *Proteobacteria*, and *Actinobacteria* are very similar to those found in healthy commensals [100].

Preclinical research is increasingly demonstrating compelling evidence and agreement that microorganisms in the gut influence many beneficial functions in humans. Furthermore, Ammons et al. conducted research on the presence of microbes in diabetic patients [100] and studies have expanded its concept such that, while the diversity of bacteria was independent of chronic wound type, there were more prevalent bacteria such as *S. epidermidis* identified in patients with diabetic foot ulcers, and *Pseudomonas aeruginosa* exhibited with a higher relative abundance overall in patients with chronic wounds demonstrating biofilm formation [101]. 

Many other studies were conducted to determine the types of microbes found in DFU. In general, three to five species of microorganisms are identified in an infected DFU, which consists primarily of Gram-positive aerobes (*Staphylococcus aureus*, *Staphylococcus epidermidis*, *Corynebacterium* spp.); Gram-positive anaerobes (*Enterococcus* spp., *Propionibacterium* spp., *Streptococcus* spp., *Peptostreptococcus* spp., *Peptococcus* spp.); Gram-negative aerobic microbes (*Pseudomonas aeruginosa*, *Acinetobacter* spp.); Gram-negative anaerobes (*Proteus mirabilis*, *Escherichia coli*, *Bacteroides* spp.); and fungi including the *Candida* spp. [102,103]. It also depicted a higher prevalence of Gram-negative pathogens in low-income countries; the most common bacteria observed in DFU is *Pseudomonas aeruginosa* [104,105].

A 16SrDNA pyrosequencing was performed by Wolcott et al. [32]. There were 910 patients with chronic diabetic foot ulcers among the total of 2963 patients. The results of this study show that diabetic patients’ chronic wounds had a significantly higher prevalence of *Staphylococcus* species. Furthermore, there was a high abundance of *Pseudomonas* species in the chronic wound samples, which included a variety of other species such as *P. aeruginosa*. However, *Corynebacterium*—a traditional commensal that made up more than 1% of the total bacterial population in more than one-third of the samples. Despite the fact that chronic cutaneous wounds are subjected to relatively high levels of oxygenation, a large number of anaerobic bacteria were found in the wound samples. *Finegoldia* spp. were found in 25% of the wounds, while *Prevotella* spp., *Peptoniphilus* spp., and *Anaerococcus* spp. were found in 12, 16, and 18% of the wounds respectively [32]. 

Multiple independent, culture-based studies found that Gram-positive cocci (GPC) are the most consistently isolated microbes from DFU patients. Furthermore, *Staphylococcus aureus* is the most commonly observed species, accounting for more than 50% of all wounds, followed by coagulase-negative *Staphylococci* spp. and *Streptococcus* spp. [106,107,108,109]. More evidence suggests that *Staphylococcus* spp. and *Corynebacterium* spp. are common in wounds, followed by a plethora of diverse anaerobic communities in DFU patients [110,111]. In the study conducted by Kalan and colleagues, the most abundant genera investigated in descending order were *Staphylococcus* (18.95%), *Corynebacterium* (14.64%), *Pseudomonas* (9.37%), and *Streptococcus* (7.32%) [111]. 

**Figure 2:**
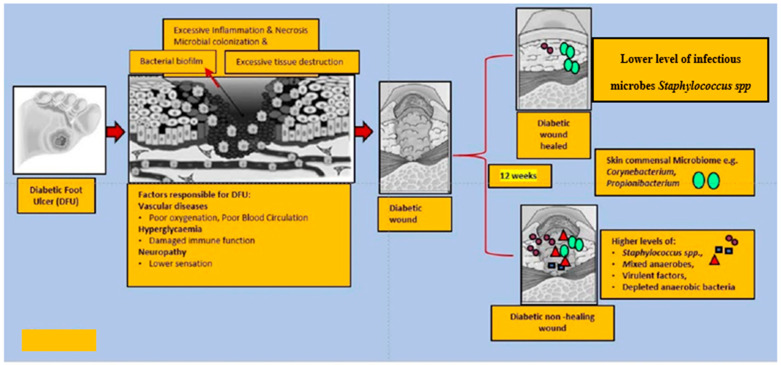
This figure explains the altered microbiota in diabetic wound healing. In general, diabetic foot wounds are complicated by various factors contributing to impaired tissue regeneration. Several factors impairing wound healing and associated factors are hyperglycaemia, peripheral neuropathy, vascular disease, and a complex microbiome. It is challenging to identify microbial communities that assemble in wound tissue and have not necessarily been associated with cardinal signs of infection. A debridement elicited reduced diversity of bacteria, governed by decreased anaerobic bacterial abundance in the overall community. One subset of wounds achieved complete re-epithelialization within 12 weeks. Kalan et al. [111] investigated the role of colonizing microbiota in wound healing, clinical outcomes, and a response to therapy in patients with chronic diabetic wounds. Strains of the wound pathogen S. aureus were associated with poor outcomes, and sharp debridement therapy depleted anaerobic bacteria in wounds with favorable outcomes.

**Figure 3:**
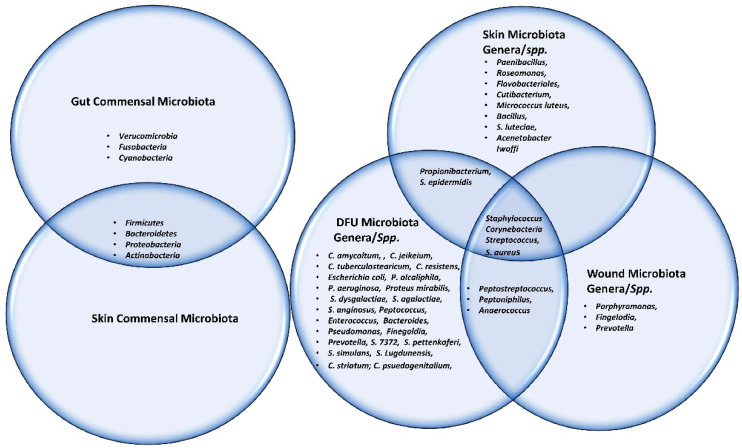
This Venn diagram depicts all the microbes (commensal, genera as well as spp.) present in gut, skin, wound and DFU.

The Shotgun metagenomics analysis from the DFU patients showed *S. aureus* as the major *Staphylococcus* species and was dominated by a single strain, *S. aureus* 7372, from *Staphylococcal* species present in lesser abundance included the coagulase-negative species such as *S. pettenkoferi*, *S. epidermidis*, *S. simulans*, and *S. lugdunensis*. *Corynebacterium striatum*, a bacterium that has been associated with infection and multi-drug resistance [112], was the most prevalent *Corynebacterium* spp. classified in DFU and showed a positive correlation with ulcer duration, while *C. jeikeium*, *C. amycolatum*, *C. pseudogenitalium*, *C. tuberculostearicum*, and *C. resistens* were present in lesser abundances. *Pseudomonas* spp. were the third most abundant genera detected, with the most abundant species identified as *P. aeruginosa* followed by *P. alcaliphila*. *P. aeruginosa* that is a commonly known pathogen associated with DFU as it is frequently isolated by culture-based methods. *Streptococcus* was the fourth most abundant genera, with *S. agalactiae*, *S. dysgalactiae*, and *S. anginosus* present in patients with DFU [111]. Many experiments have been conducted in recent years by various groups of scientists to better understand the role of microbiota in the wounds of DFU patients. According to some studies, when biofilm occurs in DFU patients, the most abundant components observed are various species of *Staphylococcus* as well as some diverse anaerobes; some groups of scientists also reported the presence of *Pseudomonas aeruginosa* as prevalent in DFU patients. We summarized the observed microbes in the gut, skin, wounds, and DFU mentioned in the manuscript, in Table 1. Based on existing knowledge of wound microbiota in DFU patients, the higher or lower abundance of microbes such as various strains of *Staphylococcus* spp. with some other anaerobes mentioned above may enable clinicians and scientists to make a thorough diagnosis of individual wounds, which may lead to improved patient prognoses through the selection of optimal treatment strategies that could be used in hospitals. Figure 2 describes the altered microbiota in diabetic wound healing [111,113] and Figure 3 describes the observed microbiota involved in skin, wounds, and DFU.

## 7. Probiotics Therapy in Diabetic Wound Healing

Because probiotics are live microorganisms, they are non-pathogenic bacterial strains that have many beneficial effects, such as improvement of normal gastrointestinal microbiota in the host, particularly when consumed in required proportions [24]. Increased evidence that is associated with the use of probiotics in wound healing and infection in various diseases, including DFU, has emerged in recent years [24]. 

Choundappan and group [114] showed that local administration of probiotics, specifically *Lactobacillus Plantarum* (5 billion CFU) strain, improved wound healing in 36 DFU patients. In this experiment, the probiotics solution was applied to the wound at the time of dressing every day. The wound swab culture was examined on several occasions, including day 0, day 5, and day 10. The findings of this study were promising, with the number of wounds with a positive status decreasing as the course progressed in either group of patients. At the end of day 5, eight individuals in the intervention group had negative wound swab cultures, while only six individuals in the control group had negative wound swab cultures. On day 10, 12 subjects in the intervention group had negative wound cultures, whereas 10 in the control group had positive wound cultures. This study concluded that probiotics can be used safely in the treatment of infected diabetic wounds by hastening the wound healing process, as shown by a significant difference in the wound bed score on day 7 [114].

Another clinical trial using the probiotics on DFU patients was performed by Mohseni et al. [115]. The study included a randomized double-blind, placebo-controlled trial of probiotics supplementation in 60 DFU patients. The patients were divided randomly into two different groups to obtain everyday either a probiotics capsule that consisted of *Lactobacillus acidophilus*, *Lactobacillus casei*, *Lactobacillus Fermentum*, and *Bifidobacterium bifidum* (2 × 10^9^ CFU/g each) or a placebo (n = 30) for a period of 12 weeks. The outcome of the study was promising and it showed significant beneficial effects specifically on the size of ulcer, the level of glycemic control, the cholesterol, plasma nitric oxide, the total antioxidant capacity that supports the diabetes wound healing mechanism.

Mohtashami et al. [116] published a study that demonstrated the use of probiotics and their beneficial effects in the diabetes wound healing mechanism. It was performed on Wistar rats, and it claimed that the animals’ wound healing process was accelerated when compared to untreated wounds. *Lactobacillus bulgaricus* and *Lactobacillus plantarum* bacteria strains were used as probiotics in the treatment of diabetes wounds. The duration of diabetic wound healing was 94–98% approximately in 14 days observed in the probiotic treatment group, which is consistent with the duration of wound healing observed in other studies.

Campos et al. also conducted a study to assess the impact of perioperative probiotic administration on the cutaneous healing process in diabetic rats. The rats in this study were given the Probiatop and were given probiotics (P) orally [117]. According to the study conducted by Mohseni et al., the probiotic is primarily composed of a mixture of four different strains of bacteria such as *Lactobacillus paracasei* LPC-37, *Bifidobacterium lac-tis* HN0019, *Lactobacillus rhamnosus* HN001, and *Lactobacillus acidophilus* NCFM at doses of 1 × 10^9^ CFU/g [115]. The experimental design consisted of two distinct groups—groups were given a probiotic mixture or maltodextrin for a continuous five days prior to the creation of the skin excisional wound. Consumption was continued until the day of euthanasia. The promising result revealed that peri-operative probiotic supplementation in diabetic rats promotes improved skin healing, attenuation of the inflammatory response, accelerated wound neovascularization, increased wound type I collagen deposition, and weight loss prevention. Glycemic control in the animals was shown to be improved. *Lactobacilli*’s beneficial effect was tested on a mouse model, where *Lactobacilli* bacteria were transformed into CXCL12-producing vectors to bioengineer the wound microenvironment after topical application. *Lactobacillus reuteri*, which expressed CXCL12, stimulated immune cells. The healing process is propelled by immune cells [118]. Overall, the frequent communication within the gut–brain–skin axis may represent a strong link between the gut microbiome and cutaneous health. However, these connections, as well as the exact mechanism involved, are still poorly understood. Probiotics may provide a potentially beneficial therapeutic approach that can safely alter the gut–skin axis and modify systemic health in patients with wound healing disorders. Furthermore, it is necessary to comprehend the interaction between the host’s respective pathways and the beneficial microbiota. It would also be beneficial to describe in detail the therapeutic potential of topical probiotics and how beneficial bacteria could alter the gut-skin axis in modifying systemic health in patients suffering from various disorders. Given the increased research on probiotics and the important role they play in human health, their use as an integrative treatment opens up a new avenue for treating patients with wound healing disorders [119]. The review published by Wang et al. [120] summarizes the possible link between gut microbial flora, probiotics, and diabetes, concentrating on the procedure through which probiotics relieved diabetes explicitly by targeting intestinal microbiota from different aspects of oxidative stress, immune responses, amino acid metabolism, intestinal permeability, and short-chain fatty acids (SCFA). Overall, the findings of the study have laid the groundwork for future clinical research and development efforts to identify a possible group of microbes with anti-diabetes effects that can be used as probiotics to improve intestinal homeostasis and alleviate metabolic diseases such as diabetes. These effects, however, have only been determined for *Lactobacillus* microbial species at this time. Further research is needed to investigate the range of effective bacterial strains that can be used as probiotics to lower glucose levels in diabetes and could be another factor to consider in the prevention of chronic wounds and thus facilitate faster wound healing in DFU [121]. However, the causal relationship between an imbalance in intestinal flora and diabetes, as well as the underlying mechanism(s), has not been fully established; further clinical trials in DFU patients are required.

It is not uncommon for external or internal factors to alter the balance between the skin and skin microbiome causing skin disorders, infection, and impaired wound healing. A wound’s microbes and pathogens are exposed to a broad range of microenvironments during wound formation and healing. As wounds heal, microenvironments increasingly change. Therefore, microbes respond physiologically to enhance the host’s innate immune system or to prevent pathogenic infection from the primary or opportunistic pathogens. Stress suppresses the production and localization of AMP, impairs barrier permeability, and increases susceptibility to infection; researchers have already published evidence to support this conclusion. It is possible that [122,123] may delay wound healing, including DFU. Based on the description given in this review, supplementation with beneficial microbes, e.g., probiotics, during stressful times or in the cases of skin dysbiosis may promote wound healing.

In this review, we discussed how probiotics, both orally and topically delivered, influence wound healing in DFU. Probiotics are known to aid in wound healing by stimulating the production of immune cells, and they also have antagonistic effects against pathogens via competitive exclusion of pathogens [124]. According to a recent publication [27], the skin and gut have different morphologies but share some physiological characteristics. Interactions between the gut and skin are centered on microbiota and metabolites secreted by them, which can interfere with biological processes regulating metabolism, immunity, inflammation, oxidative stress, and neuroendocrine function. The mechanism of action by which gut health affects skin health (from the inside out) is critical for defining cross-communication between the two compartments. The finding presented an essential aspect of gut microbiota, skin homeostasis, and skin wound healing with probiotics during the gut-skin axis.This discovery revealed an important aspect of gut microbiota, skin homeostasis, and skin wound healing with probiotics within the gut-skin axis. Based on the aforementioned studies, this review also supports the role of the gut-skin axis in wound healing in DFU. Therefore, based on the currently available literature, well-designed clinical trials, systematic review results, and various experimental findings, it would be ideal for clinical doctors and researchers to focus on clinical trials specifically targeting DFU patients to investigate the influence of probiotics on wound healing. It also necessitates understanding the role of beneficial bacterial strains in wound healing mechanisms, identifying the strain, determining the optimal dose, and determining the duration of perioperative supplementation. As a result, the use of these bacterial strain mixtures found in probiotics can be regarded as a challenging therapeutic approach for the treatment of diabetic wounds. Table 2 presents a summary of probiotics used in DFU treatment in last 10 years.

## 8. Conclusions and Future Directions

As the global population of diabetics expands, so does the incidence of chronic nonhealing wounds, putting a significant strain on physicians, the healthcare system, and society. The whole wound healing mechanism is intricate and well-coordinated, and if it is disturbed, it can be disastrous. The bacteria in a wound bed, when combined with many host variables, can stymie wound healing and result in delayed, chronic wounds. The wound microbiota is thought to remain a biofilm in chronic wounds, resistant to antibiotics and mechanical treatments.

Probiotics have shown efficacy in a variety of human and animal models for improving many aspects of wound healing in diabetic patients, but there are still many unsolved problems. We hope that this review will prompt the start of well-conducted prospective studies to determine the role that probiotics could play in allowing for efficient, safe, and reproducible wound healing, as well as potential clinical trials.

Even with significant advances in microbiome research, it remains difficult; we need more and more studies to determine how the microbiome influences wound healing and vice versa. Ultimately, the goal is to use the knowledge gained of the skin microbiome to promote the healing of acute and chronic wounds in diabetes patients. Finally, the modifiable effects of the human gut microbiota on the development of metabolic syndrome make its manipulation a promising therapeutic approach. Furthermore, more detailed insights using a complementary method for bacterial identification and well-defined populations with diabetic foot ulcers in conjunction with standardized sampling methods may be a better option to understand bacterial diversity, which may provide new insights to redirect treatments in clinical practice to improve potential outcomes in the future. Furthermore, the microbiome is a therapeutic target that can be modified; a fundamental understanding of its components will reveal novel targets for managing and treating diabetic wounds. Based on the discussion in this review, additional clinical trials to confirm the potential benefits of probiotic treatment for managing chronic wound healing in diabetes should be conducted. The addition of probiotics to current therapeutics may improve wound healing, aid in recovery, and help suppress pathogen invasion by accelerating a higher volume of *Pseudomonas* spp. and *Staphylococcus* spp. in the skin microbiome, as any change in the skin commensal exhibited a significant impact on the cutaneous wound healing process. Furthermore, with its central role for microbiota, the gut-skin axis represents an exciting field of research with a wide range of therapeutic applications, including diabetes wound healing. Moreover, an in-depth understanding of the complex mechanism for the gut–skin axis is required, as well as a unified methodological approach to resolve wound healing in DFU patients. In addition, a recent study published by Tembhre et al., 2022 supported the idea that probiotics are beneficial in the mechanism of wound healing.

## Figures and Tables

**Table 1:** Summary: Commensal microbes, Various genera and microbial species observed in gut, skin, wound and DFU.

Gut Microbiota	Reference	Skin Microbiota	Reference	Skin Microbiota	Reference	Wound Microbiota	Reference	DFU Microbiota	References
Commensal		Commensal		Genera/spp.		Genera/spp.		Genera/spp.	
*Firmicutes*	[6,7]	*Actinobacteria*	[22]	*Cutibacterium*	[22,44,57,61]	*Staphylococcus*	[94,95]	*Staphylococcus*	[35,98,102,107,108,109,110,111]
*Bacteroidetes*				*Micrococcus luteus*		*S. aureus*		*S. aureus*	[35,102,105,106,112,113,114]
*Proteobacteria*		*Firmicutes*	[22]	*Staphylococcus*		*Corynebacterium*		*S. 7372*	[103,104,113]
*Actinobacteria*		*Proteobacteria*	[22]	*S. aureus*		*Porphyromonas*		*S. pettenkoferi*	
*Verucomicrobia*				*S. epidermidis*		*Streptococcus*		*S. epidermidis*	
*Fusobacteria*				*Streptococcus*		*Fingelodia*	[96]	*S. simulans*	
*Cyanobacteria*				*S. luteciae*		*Prevotella*		*S. Lugdunensis*	
Intestinal Lumen				*Bacillus*,		*Peptonipihlus*		*Corynebacterium*	[35,98,103,104,111,113]
*Bifidobacteria*	[10]			*Paenibacillus*		*Peptostreptococcus*		*C. striatum*	[113]
*Bacterodes*		*Bacteroidetes*	[96]	*Roseomonas*		*Anaerococcus*		*C. jeikeium*	[112]
*Prevottela*				*Flavobacteriales*				*C. amycoltum*	
*Lactobacillus*				*Corynebacteria*	[44,57,96]			*C. psuedogenitalium*	
*Clostridium*				*Propionibacterium*				*C. tuberculostearicum*	
*Streptococcus*				*Acenetobacter Iwoffi*				*C. resistens*	
*Enterococcus*								*Pseudomonas*	
*Ruminococcus*								*P. alcaliphila*	
Mucosa-associated surface								*P. aeruginosa.*	
*Clostridium*								*Streptococcus*	[103,104,107,110,112,113]
*Lactobacillus*								*S. dysgalactiae*	[112]
*Enterococcus*								*S. agalactiae*	
*Akkermansia*								*S. anginosus*	
								*Finegoldia*	[35]
								*Prevotella*	
								*Peptoniphilus*	[35,103,104]
								*Anaerococcus*	[35]
								*Enterococcus*	[103,104]
								*Propionibacterium*	
								*Proteus mirabilis*	
								*Escherichia coli*	
								*Bacteroides*	
								*Peptococcus*	
								*Peptostreptococcus*	

**Table 2:** Lists of probiotics used in DFU treatment.

Samples	Probiotics	Mode of Probiotic Administration	Outcome	References
DFU patients	*Lactobacillus Plantarum*	Solution applied on wound during dressing	Improved wound healing	[114]
DFU patients	*Lactobacillus acidophilus, Lactobacillus casei, Lactobacillus Fermentum*, and *Bifidobacterium bifidum*	Probiotic capsule	Reduced DFU size	[115]
Wistar rats	*L*. *bulgaricus* and *L. plantarum*	Probiotic administration on wound	Improved wound healing	[116]
Wistar rats	*Lactobacillus paracasei* LPC-37, *Bifidobacterium lactis* HN0019, *Lactobacillus rhamnosus* HN001 and *Lactobacillus acidophilus* NCFM	Oral administration of Probiotics	Improved wound healing	[117]
C57BL/6 Mice	CXCL12-expressing *Lactobacillus reuteri*	Administered centrally in Wound	accelerated wound closure in in mouse	[118]

## Data Availability

Not applicable.

## Abbreviations

DFU: Diabetic Foot Ulcers; ECM: extracellular matrix; FEP: functionally equivalent pathogroups; EC: epithelial cells; GALT: gut-associated lymphoid tissue; SCFA: short-chain fatty acids; AMP: antimicrobial proteins
